# Role of PCDH 1 Gene in the Development of Childhood Asthma and Other Related Phenotypes: A Literature Review

**DOI:** 10.7759/cureus.3360

**Published:** 2018-09-25

**Authors:** Sharmi Biswas

**Affiliations:** 1 Pediatrics, California Institute of Behavioral Neurosciences & Psychology, Fairfield, USA

**Keywords:** asthma, rhinitis, eczema, atopic dermatitis, pcdh 1, epithelium, cadherin

## Abstract

The asthma gene PCDH 1, encoding protocadherin-1, is a cellular adhesion molecule which plays an important role in epithelial barrier formation and repair. PCDH 1 is a novel susceptible gene not only in childhood asthma but also in eczema and other atopic phenotypes. In this article, we reviewed relevant articles from PubMed, Google Scholar, Science Direct and included all available significant pieces of information about the PCDH 1 association with asthma and other atopic or non-atopic phenotypes. It is very interesting that cigarette smoking can induce changes in PCDH 1 expression but how the changes in PCDH 1 induce asthma is still not clear. PCDH 1 gene polymorphism also sometimes plays role in asthma and bronchial hyperresponsiveness (BHR) pathogenesis as well as in allergic dermatitis.

## Introduction and background

Childhood asthma is one of the most common causes of emergency department visits, hospitalizations and absence in schools. As a disease with increasing incidence in children, asthma became an area to focus on recent clinical research. Asthma is a multifactorial disease including environmental and genetic factors. Bronchial hyperresponsiveness (BHR) develops from chronic pulmonary inflammation leading to recurrent episodes of wheezing, chest tightness, cough at night or in the early morning. The variable airflow in the lungs is reversible spontaneously or with treatment. Different respiratory phenotypic subtypes in children are associated with different risk factors. Some related genes had been discovered in recent years which play a crucial role in the pathophysiology of childhood asthma [1]. Protocadherin-1 (PCDH 1) gene is recently got identified as a susceptible gene to be responsible for developing bronchial hyperresponsiveness and asthma. The location of the PCDH 1 is in 5q 31-33 chromosome which is the harboring zone for several genes like Interleukin-4 (IL-4), Interleukin-5 (IL-5), Interleukin-13 (IL-13), the candidate genes for asthma and allergy traits [2, 3].

Most commonly children with asthma also suffer from other atopic diseases like atopic dermatitis which indicate the common environmental and genetic backgrounds shared by asthma and atopic disease. Genetic variation in epithelial cell barrier and repair function is the very significant factor to develop asthma and atopic dermatitis [1]. PCDH 1 works in the cell to cell adhesion. PCDH 1 mRNA are found both in skin keratinocytes and in respiratory epitheliums. A recent study showed that the PCDH 1 gene has a pleiotropic effect on BHR and eczema in the different population [4, 5].

## Review

Protocadherin expression in the body

**Figure 1:**
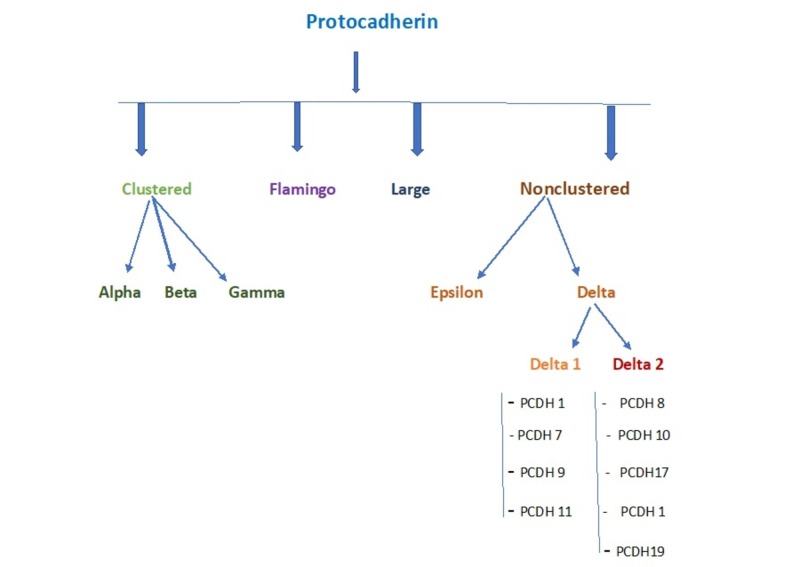
Types of protocadherins.

Protocadherins are the largest cadherin subfamily of the cell adhesion molecules. The classification of protocadherin is shown in Figure 1.

Delta cadherins have a different structure than classic cadherins due to the presence of seven (Delta 1) and six (Delta 2) extracellular cadherin repeats and variable intracellular signaling domains. Clustered protocadherins express only in the nervous system while non-clustered delta 1 protocadherins express widely in different types of tissues. PCDH 7 is responsible for non-small cell lung cancer, PCDH 9 in autism spectrum disorder and PCDH 11 in Alzheimer’s disease. The recent study identified PCDH 1 as the susceptible gene for asthma. Delta 1 subfamily possesses three conserved motifs (CM 1, 2 and 3) in the intracellular cytoplasmic tail. CM 3, which plays role in lung morphogenesis, is exclusively present in only delta 1 protocadherin subfamily. PCDH 1 shows homotypic adhesion function in cells, but weaker than classic protocadherins. A study proved that PCDH 1 has higher expression in terminally differentiated bronchial cells than the undifferentiated cells which signify the potential role of PCDH 1 in the pathogenesis of asthma. Abnormal regulation of PCDH 1 expression leads to slower or incomplete differentiation of the epithelial layer, eventually causing the dysregulated response to injury and weaker epithelial barrier function [6]. Kozu et al., in 2015, found that PCDH 1 has an important role in the physical barrier in airway epithelium. A PCDH 1 expression is upregulated by corticosteroid use which is an interesting finding to explain the role of PCDH 1 in asthma [7].

PCDH 1 mRNA is expressed in brain, skin, lung, nose and also throughout all epithelial and endothelial lineages during embryonic mouse development. In a skin keratinocyte wounding model, PCDH 1 gene expression is upregulated, indicating the crucial role of PCDH 1 in epithelial barrier function [3, 5]. Barrier dysfunction is also responsible for the development of other atopic diseases like atopic dermatitis, eczema, and rhinitis [8]. Three PCDH 1 polymorphisms (rs3797054, IVS3-116, rs38223570 have been detected to be related with BHR and asthma in several cohort studies done in the USA, the UK and Dutch populations). In two independent Dutch birth cohort, a relation between PCDH 1 polymorphism IVS3-116 and eczema was detected [5].

Aetiologies of childhood asthma

Asthma is a genetically heterogeneous disease. Genome-wide association study (GWAS) recently identified a strong association between childhood asthma (wheezy phenotype) and a specific locus on chromosome 17q21 (which encodes ORMDL3 and GSDMB). Chromosome 17q21 is closely associated with the development of recurrent wheeze, asthma exacerbation, and bronchial hyperresponsiveness but no relation with eczema, rhinitis or allergic sensitization. As there was no evidence of atopy in the association of 17q21 and the respiratory symptoms, it can be accepted that 17q21 is an important risk factor for childhood non-atopic asthma. FCER1A is the gene which shows high affinity for the IgE receptors, may influence the serum IgE levels. But polymorphism in FcER1A gene changes serum IgE level in both asthmatic and non-asthmatic patients. Polymorphism in PCDH 1 gene and PDCD 4 gene is also the responsible factor for severe childhood and non-atopic asthma. Several other associated loci with asthma risk are DENND1B, IL1RL1, IL18R1, HLA DQ, IL33, IL2RB, and SMAD3. An SNP rs4950928, in the promoter region of CHI3L 1 expression in the airway epithelium, is also associated with childhood asthma [9-10].

Another common risk factor for childhood asthma is maternal asthma and child’s sex. Maternal asthma and male child indicate the higher risk of developing childhood asthma while female gender and paternal asthma is significantly associated with adult-onset asthma. Maternal microchimerism (non-self cells in the blood of an individual) is one of the possible factors which provide protection against childhood asthma. Presence of maternal cells in the child is called maternal microchimerism. Chimeric cells have stem cell-like properties and they can be differentiated to perform immunological as well as multiple other functions [11].

Some environmental factors also contribute to the development of childhood asthma such as air pollution, maternal smoking, diet and microbial exposure in early life like rhinovirus infection which causes decreased methylation in DNA at multiple CpG sites. Interestingly a study found a close connection between adverse childhood experience (ACE) and immunological dysregulation and childhood-onset asthma and other diseases. Even perinatal maternal stress is a causative factor for early-onset asthma in the offspring due to placenta-induced secretion of the corticotropin-releasing hormone, activating the fetal hypothalamic-pituitary-adrenocortical axis and eventually leading to dysregulation of glucocorticoid desensitization. This pathophysiology is also responsible for other atopic diseases like eczema, food allergies [12].

A study showed that PCDH20 gene, which codes protocadherin-20 (a protein involved in cell adhesion and signal transduction), has higher methylation rate in the sputum cells of asthmatic patients. To determine the epigenetic state of asthma, both environmental and genetic factors play a crucial role. It is also believed that intrauterine and early life exposure to a farm animal reduces the risk of allergic disorders and asthma in children in their later life. Gestational exposure to cigarette smoking causes differential methylation of cytochrome P450 aryl-hydrocarbon-hydroxylase (CYP1A1 ) gene and aryl hydrocarbon receptor repressor gene (AHRR). Early life DNA methylation causes developing cell memory to metabolize xenobiotics differently which make children vulnerable to have an increased risk of asthma in later life [13].

Pathophysiology of asthma

**Figure 2:**
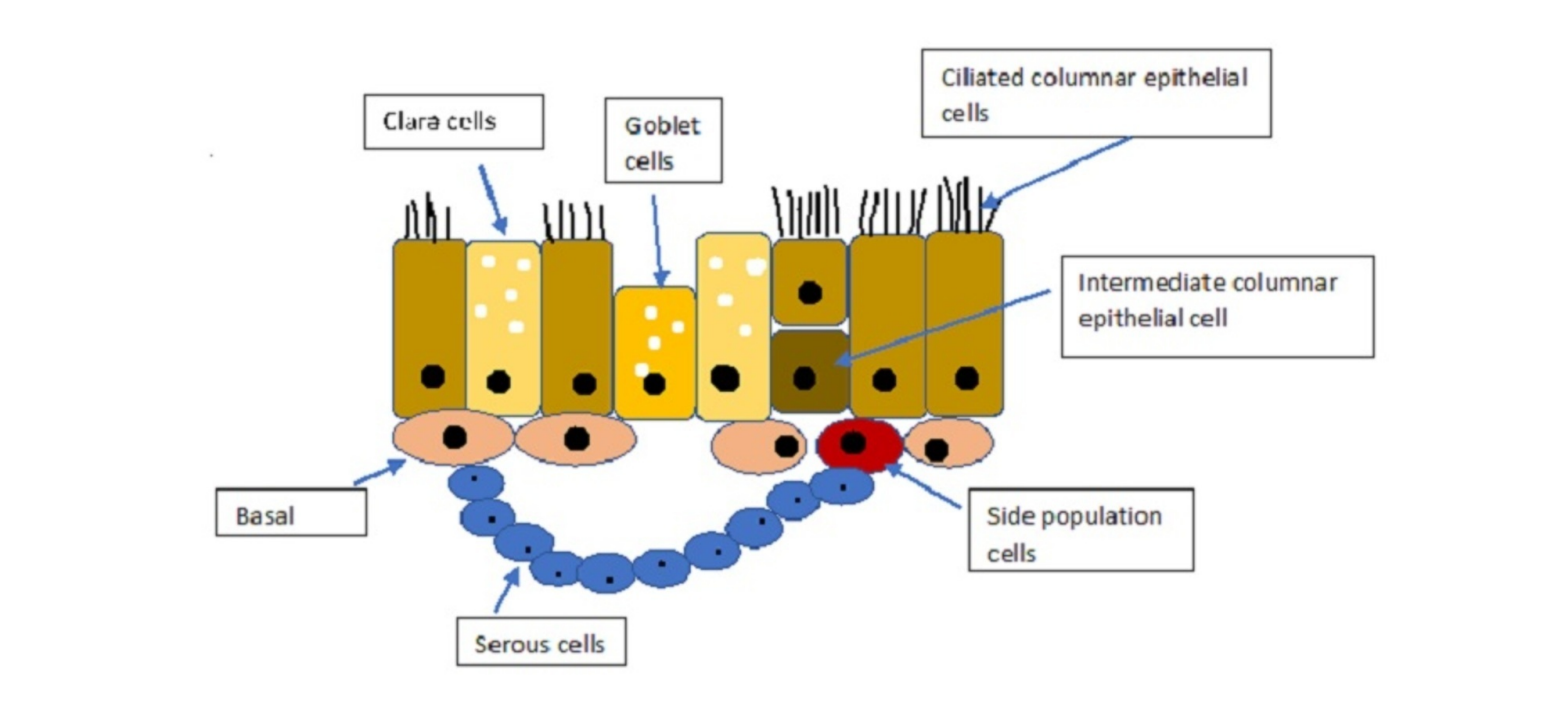
Structure of respiratory epithelium.

The common triggers of asthma are allergens or some foreign pathogens. Most initially, there is constriction of airway smooth muscles and inflammation in the airways (edema, inflammatory cell infiltrations and increased airway secretions). The airway epithelium is the first line defense line in-between the external environment and the submucosa. The conductive portion of airway epithelium is pseudostratified with ciliated columnar epithelial cells, goblet cells, intermediate columnar epithelial cells, populations cells, basal cells and serous cells (Figure 2) [10, 11, 14-16].

Several structural and functional abnormalities are seen in asthmatic airway epithelium-like enormous resident stem cells and basal cells, hyperplasia of goblet cells and excessive mucin production along with the reduced number of ciliated cells which indicate dysregulation of cell differentiation. Changes in epithelium increase the risk of oxidative stress, release of cytokines and extracellular matrix, synchronization of mitosis and deficiency of innate immunity which subsequently cause the abnormalities in epithelial barrier function and repair process [10, 14-16].

PCDH 1 gene in asthma and eczema

Genetic variants in the encoding gene of PCDH 1 have been identified to be associated with asthma, eczema, and wheeze in the early childhood. Passive smoking in early childhood is responsible for gene-environment reaction causing the change in PCDH 1 gene to develop asthma. Genetic association studies showed that PCDH 1 gene is associated with BHR, eczema, nonatopic childhood asthma, and transient early wheeze. PCDH 1 dysfunction induces the defect in epithelial barrier function which is significant in asthma and eczema [9].

In a Dutch study, there was an observed association between PCDH 1 polymorphism IVS3-116 and eczema BHR and asthma [5]. Strong relation discovered between PCDH 1 gene with asthma and BHR in the population exposed to environmental tobacco smoke. Exposure to tobacco smoke induced robust reduction of PCDH 1 mRNA expression in mouse airway epithelium. This downregulation sustained even after two months of cigarette smoking exposure [16]. PCDH 1 is mainly expressed in the ciliated epithelial cells of the airway and nasal epithelium. In patients with asthma and chronic rhinosinusitis, PCDH 1 expression was reduced in the airway regions containing inflammatory cells, epithelial detachment, and wide intercellular space, indicating low PCDH 1 expression is associated with increased damage and vulnerability of epithelial barrier function [17]. Mutation of the PCDH 1 gene also induces allergic asthma as barrier dysfunction may change the response to antigen exposure. PCDH 1 possessing chromosome 5q31-33 has been identified as the responsible gene for BHR and atopy. 5q 31-33 chromosome is the major locus regulating serum IgE level. Bronchial hyperresponsiveness is a significant risk factor for asthma. Bronchial hyperresponsiveness and elevated total serum IgE level are co-inherited but all the factors related to asthma are not related to elevated IgE levels [18]. Glucocorticoid is the most effective current therapy for bronchial asthma. It works by improving the epithelial barrier function. PCDH 1 isoform 2 expression is getting increased by induction of glucocorticoid. Isoform 2 has an ameliorating effect on epithelial barrier function, which signifies increased epithelial barrier function and PCDH 1 expression. An important transcription factor NF-E2 related factor (Nrf2), which protects cells from antioxidant stress, also has a significant role in the stabilization of airway epithelium following glucocorticoid exposure [7, 19].

## Conclusions

Though childhood asthma is a common disease in a pediatric population, very few studies are available to provide in-depth information on genetic association with it. The role of PCDH 1 is explained by several studies. But still, more human studies with large sample size should be done to know about the accurate role of PCDH 1 in asthma and other childhood atopic phenotypes. Detection of PCDH 1 role in the pathophysiology of childhood asthma will not only help to reveal the accurate prevention strategy of childhood asthma but it will also help to do further research for an invention of molecular-targeted therapy. There is a big question how cigarette smoke can induce changes in PCDH 1 and whether other environmental pollution like engine smoke, dust, pollen exposure also can induce a change in PCDH 1 expression in the airway epithelium.
